# Metastasis patterns and prognosis in breast cancer patients aged ≥ 80 years: a SEER database analysis

**DOI:** 10.7150/jca.63813

**Published:** 2021-09-03

**Authors:** Youming Han, Zhilin Sui, Yongsheng Jia, Hailong Wang, Yan Dong, Hongdian Zhang, Zhigang Li, Zhentao Yu

**Affiliations:** 1 Department of Esophageal Cancer, Tianjin Medical University Cancer Institute and Hospital, National Clinical Research Center for Cancer, Key Laboratory of Cancer Prevention and Therapy, Tianjin's Clinical Research Center for Cancer, Tianjin 300060, China.; 2 Department of Respiratory medicine, Binhai Hospital of Tianjin Medical University General Hospital, Tianjin, 300456, China.; 3 Department of Breast Oncology, Tianjin Medical University Cancer Institute and Hospital, Tianjin, 300060, China.; 4 Tianjin Teda Hospital, Tianjin, 300456, China.; 5 Department of Thoracic Surgery, National Cancer Center, National Clinical Research Center for Cancer, Cancer Hospital & Shenzhen Hospital, Chinese Academy of Medical Sciences and PeKing Union Medical College, Shenzhen, 518116, China.

**Keywords:** breast cancer, elderly patients, age-related, metastasis, prognosis

## Abstract

**Background:** This study aimed to investigate the metastasis patterns and prognosis of breast cancer (BC) in patients aged ≥ 80 years with distant metastases, as the current literature lacks studies in this population.

**Methods:** A retrospective, population-based study using data from the Surveillance, Epidemiology, and End Results (SEER) database was conducted to evaluate 36,203 patients with BC from 2010 to 2016. Patients were classified into three groups, the older group (aged ≥ 80 years), middle-aged group (aged 60-79 years), and younger group (aged < 60 years). The role of age at the time of BC diagnosis in metastasis patterns was investigated, and the survival of different age groups of patients with BC was assessed.

**Results:** Overall, 4,359 (12%) patients were diagnosed with BC at age ≥ 80 years, 19,688 (54%) at 60-79 years, and 12,156 (34%) at < 60 years. Compared with the other two groups, those in the older group had a lower rate of treatment acceptance. Statistical analysis revealed that older patients were more likely to have lung invasion only (odds ratio [OR]: 1.274, 95% confidence interval [CI]: 1.163-2.674) and less likely to have bone invasion only (OR: 0.704, 95% CI: 0.583-0.851), brain invasion only (OR: 0.329, 95% CI: 0.153-0.706), or multiple metastatic sites (OR: 0.361, 95% CI: 0.284-0.458) compared to the other two groups. Age at diagnosis was an independent prognostic factor for survival. The older group had the worst overall survival (OS, P<0.001) and BC-specific survival (CSS, P<0.001). Furthermore, patients aged ≥ 80 years with only liver metastasis had the worst CSS and OS.

**Conclusion:** Patients aged ≥ 80 years were less likely to be receptive to cancer-related therapy and had the highest cancer mortality rate among all patients. Our findings will hopefully help clinicians develop more appropriate modalities of cancer treatment in elderly BC patients.

## Introduction

Breast cancer (BC) is the most widely diagnosed cancer in women worldwide. In 2019, a total of 268,600 people were diagnosed with BC, and 41,488 females died of the disease 1. Survival rates in elderly patients with BC have improved in recent decades, which is largely attributed to the expanding efforts in early detection and recent advances in modalities of cancer treatment. Despite these, BC remains the second leading cause of cancer-related mortality in the United States, where 11,002 elderly patients aged ≥ 80 years were estimated to have died of BC in 2019. By 2030, nearly 20% of US population will be older adults (aged ≥ 65 years), which is one in every five persons or 70 million people 2. Metastases in organs distant from the primary site have been the main cause of mortality among patients with BC. The most common sites of metastasis include bone, brain, lungs, and liver 3.

In current practice, older patients are generally not ideal candidates for large clinical trials and might be less likely to receive treatment 4. The decline in physical function associated with aging may be the reason for the unwillingness to pursue aggressive therapy for both patients and doctors 5. A previous study showed that 61% of patients aged > 75 years with stages I to III triple-negative BC did not receive chemotherapy, in contrast to 5% of patients aged < 64 years (P < 0.001) who were declined chemotherapy. Nearly 12% of patients aged >75 years were not evaluated by an oncologist 6. Moreover, it was found that elderly individuals were less likely to receive treatment compared to young women, regardless of the cancer type. This conservative approach has shown to have a marked negative effect on BC-specific survival in older BC patients than in younger ones 7. On the contrary, another study showed some survival benefits to elderly patients with hormone receptor-positive BC who received endocrine treatment 8.

However, to our knowledge, population-based studies describing the role of age in metastatic heterogeneity of BC are limited 9. Therefore, this study aimed to investigate the metastasis patterns and prognosis of patients with BC aged ≥ 80 years.

## Material and methods

### Data collection

We used the Surveillance, Epidemiology, and End Results (SEER) database (SEER * Stat 8.3.6 version) to filter and narrow down the information to a representative population of patients for this research (http://seer.cancer.gov/). SEER is generally considered to be the gold standard for data quality among cancer registries, with near-complete case ascertainment and microscopic confirmation 10. Since this study used registry data, approval was obtained from the Ethical Committee and Institutional Review Board of Tianjin Medical University Cancer Institute and Hospital. The methods were based on approved guidelines.

### Study population

We utilized SEER population-based data to analyze distant metastasis patterns and prognosis of patients in different age groups and BC subtypes in a large cohort of the BC population, including patients aged ≥ 80 years. We limited this study population based on the following criteria: (1) age at diagnosis ≥18 years' old, (2) primary site at the breast and microscopically confirmed primary breast cancer, (3) only one malignant primary tumor, and (4) diagnosis between 2010 and 2016. Patients who did not meet these criteria were excluded. Patients were divided into three groups according to age (older group: aged ≥ 80 years; middle-aged group: aged 60-79 years; and younger group: aged < 60 years). Ultimately, a total of 36,203 patients were included.

In addition to age, other important clinicopathological parameters known to contribute individually to outcomes were also included in the analysis. The following factors were extracted: demographic factors (year of diagnosis, age at diagnosis ≥18 years old, and race), clinicopathological factors (tumor size [T stage], lymph node status [N stage]), TNM stage, histologic grade (well differentiated, moderately differentiated, poorly differentiated, undifferentiated, unknown), primary site, and morphology of ICD-O-3 (8000/8033/8010/8013/8022/8032/8035/8046/8050/8070/8071/8140/8141/8200/8201/8211/8230/8240/8246/8249/8255/8260/8310/8315/8343/8401/8480/8481/8490/8500/8501/8502/8503/8504/8507/8510/8520/8522/8523/8524/8530/8541/8543/8572/8573/8574/8575/8980/9020), breast subtype (luminal A, luminal B, triple-negative, HER2-enriched), therapeutic interventions (surgery of primary site in terms of the “breast surgery codes C50.0-C50.9”, radiotherapy, and chemotherapy), and survival factors (death events and survival months). The patients' pathological TNM stages were confirmed using the 7^th^ edition of the American Joint Committee on Cancer staging system.

### Statistical analysis

The primary outcomes were overall survival (OS) and BC-specific survival (CSS). To clarify the survival benefit of locoregional and systemic treatment for older patients, both OS and CSS of the three age groups were compared in terms of metastasis to different single organs or a combination of multiple organs. OS was defined as the time from the diagnosis of breast cancer to death due to any cause or the date of the last follow-up. CSS was calculated as the time from the diagnosis of breast cancer to death due to BC. One-way analysis of variance was utilized to compare demographic and clinical characteristics of patients among age groups. Comparison of categorical variables was performed using Pearson's chi-square test, and the Kaplan-Meier method was adopted to depict survival curves, with the log-rank test being performed to detect the differences among the curves. Univariate and multivariate Cox regression models were applied to identify risk factors for OS and BC-CSS; odds ratios (ORs) and 95% confidence intervals (CIs) were calculated. A p-value less than 0.05 was considered statistically significant. Forest plots were created using GraphPad Prism 8.2.4 (GraphPad Software, San Diego, CA, USA). All the other calculations were performed using SPSS 21.0 statistical software (SPSS Inc. Chicago, IL, USA).

## Results

### Demographics

In total, 36,203 BC patients were enrolled in the current study. Among them, 4,359 (12%) patients were diagnosed at the age of ≥ 80 years, 19,688 (54%) patients at 60-79 years, and 12,156 (34%) patients at <60 years. In the three groups, 24,560 cases (67.8%) had luminal A subtype, 2,979 cases (8.2%) had luminal B subtype, 1,418 cases (3.9%) had HER2-enriched subtype, and 5,122 cases (14.1%) had triple-negative BC. Regarding the metastasis site, 1,609 patients were diagnosed with only bone metastases, 139 with only brain metastases, 112 with only liver metastases, 252 with only lung metastases, and 1,637 with metastases to multiple sites. Statistically significant differences in clinicopathological characteristics among the different age groups with BC are summarized in Table 1. Interestingly, among the three populations, there were more white patients than any other ethnic group in the older age group (87.8% vs. 83.8% and 73.8%, respectively, P <0.001). Notably, a high proportion of the young patients had aggressive luminal A and triple-negative subtypes. The proportion of luminal B, HER2, and triple-negative subtypes was significantly higher in patients younger than 60 years (P<0.001). Furthermore, significant differences in treatment modalities were found among the different age groups. For instance, compared with the other two groups, the older group had a lower rate of treatment acceptance of surgery (86.6% vs. 91.6% vs. 87.3%, P<0.001), radiotherapy (80.1% vs. 89.3% vs. 89.1%, P<0.001), and chemotherapy (10.8% vs. 36.5% vs. 67.4%, P<0.001).

### Correlation between age and metastasis patterns of BC

**Figure 1:**
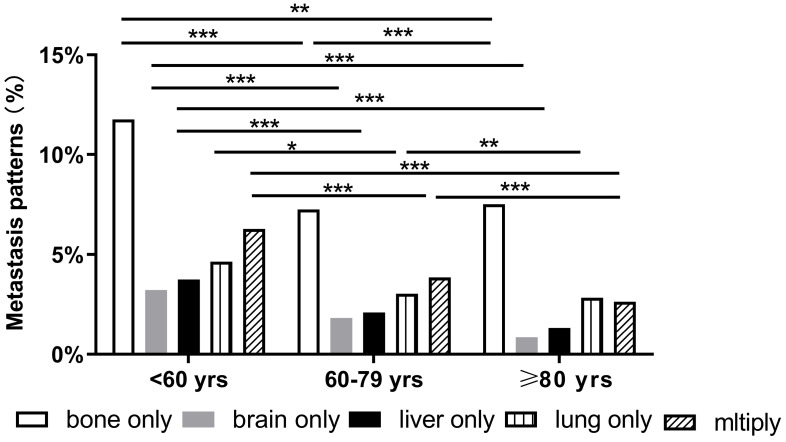
Distant metastatic patterns of breast cancer (*p < 0.05, **p < 0.01, ***p < 0.001).

**Figure 2:**
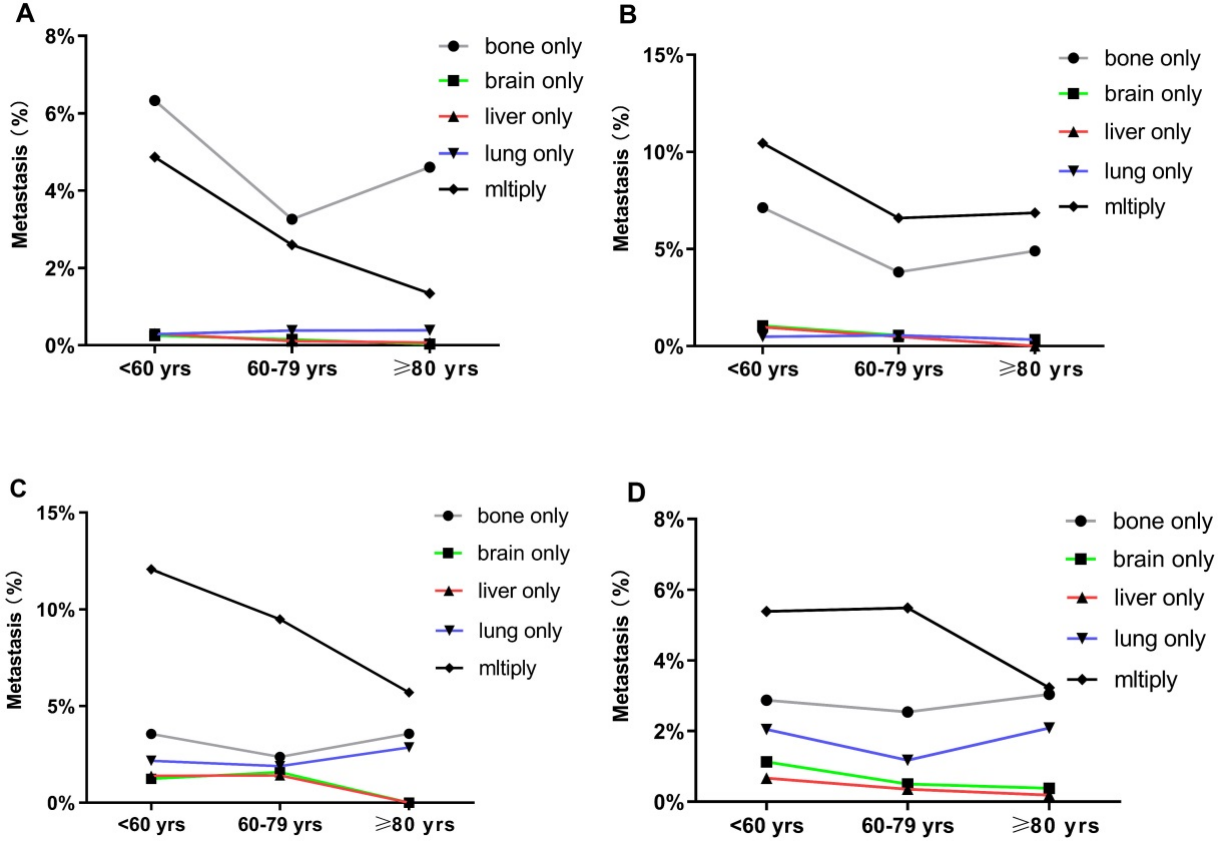
Distant metastatic patterns of breast cancer in different age groups. Metastasis patterns of (A) luminal A breast cancer; (B) luminal B breast cancer; (C) HER2-enriched breast cancer; and (D) triple-negative breast cancer were analyzed.

BC patients with single and multiorgan metastatic disease were analyzed as shown in Figure 1. Evidently, the three cohorts presented most frequently with bone metastasis and least frequently with brain and liver metastases. Compared with the other two groups, the older group showed fewer occurrences of brain-only metastatic disease (0.85% vs. 3.23% and 1.83%, respectively P<0.001). We also found that the older patients had a lower proportion of multiple metastatic sites (2.64%) than middle-aged (3.86%) and younger patients (6.28%) (P<0.001). Notably, there were some differences in the patterns of metastasis among different subtypes in the three age groups. However, in all subtypes of BC, a similar trend was that the incidence of bone metastasis was observed to be the highest in all three age groups. Compared with the other two groups, the older group had fewer occurrences of brain-only metastatic disease and liver-only metastatic disease in luminal A subtype (0.03% and 0.07%, respectively), luminal B subtype (0.33% and 0%, respectively), HER2-enriched subtype (0% and 0%, respectively), and triple-negative subtype (0.38% and 0.19%, respectively) (P<0.001) (Fig. 2).

### Risks examined for association with different metastasis sites

**Figure 3:**
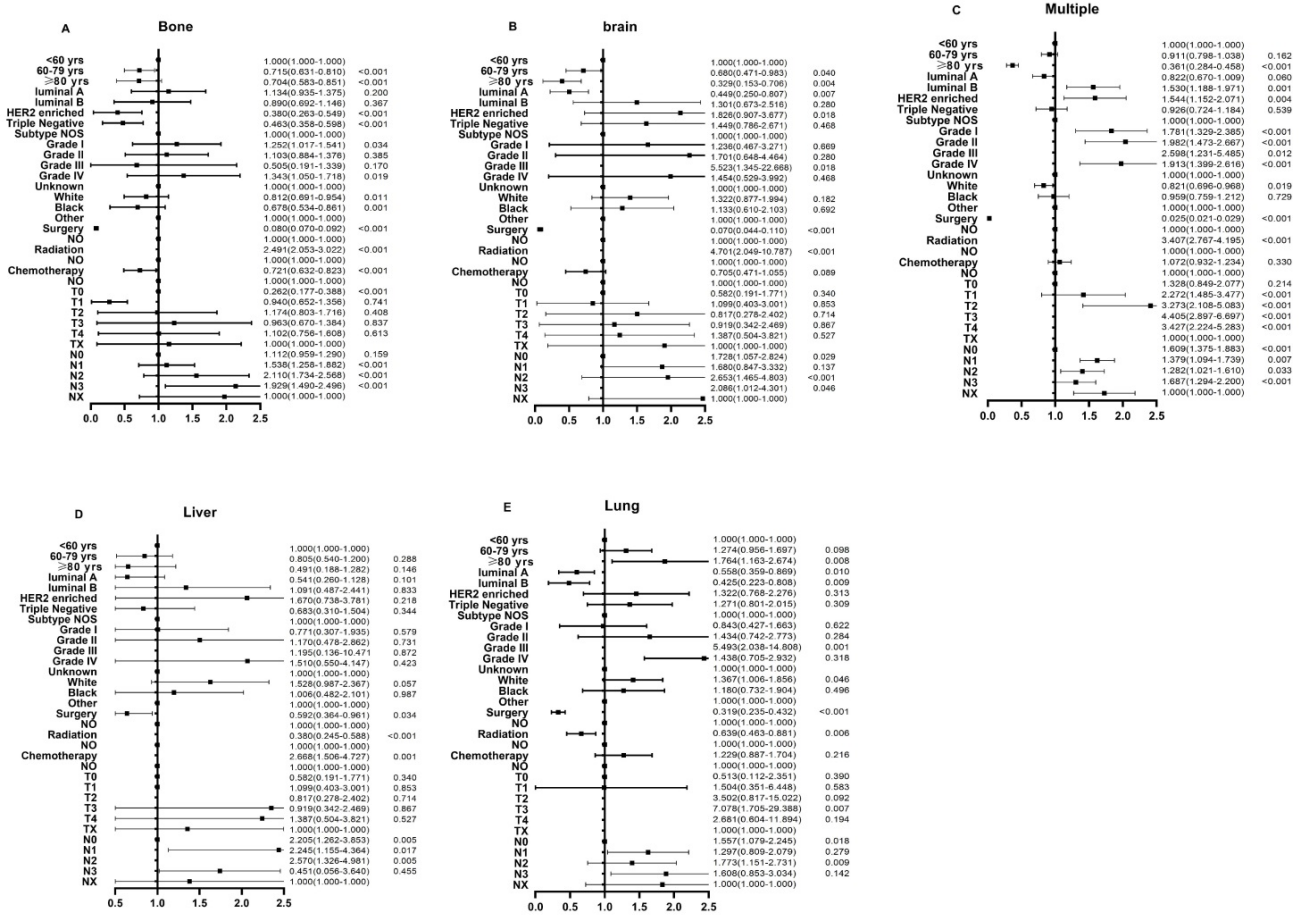
Multivariable logistic regression analyses predicting different sites of metastasis in breast cancer patients. (A) bone only metastasis; (B) brain only metastasis; (C) multiple metastatic sites; (D) liver only metastasis; and (E) lung only metastasis.

In the logistic regression models adjusted for race, subtypes, histologic grade, surgery, radiotherapy, chemotherapy, as well as T stage and N stage, the odds for bone-only metastasis (OR: 0.704, 95% CI: 0.583-0.851), brain-only metastasis (OR: 0.329, 95% CI: 0.153-0.706), and multiple sites metastasis (OR: 0.361, 95% CI: 0.284-0.458) significantly decreased in the older groups compared to those in the younger group (Fig. 3A-C). Additionally, there was no significant difference in odds for liver-only metastasis among the three groups (OR: 0.805, 95% CI: 0.540-1.200 for middle-aged group, OR: 0.491, 95% CI: 0.188-1.282 for older group, p > 0.05) (Fig. 3D). On the other hand, the older patients had higher odds for lung-only metastasis than the younger patients (OR: 1.274, 95% CI: 1.163-2.674) (Fig. 3E).

### Survival outcomes among age groups

**Figure 4:**
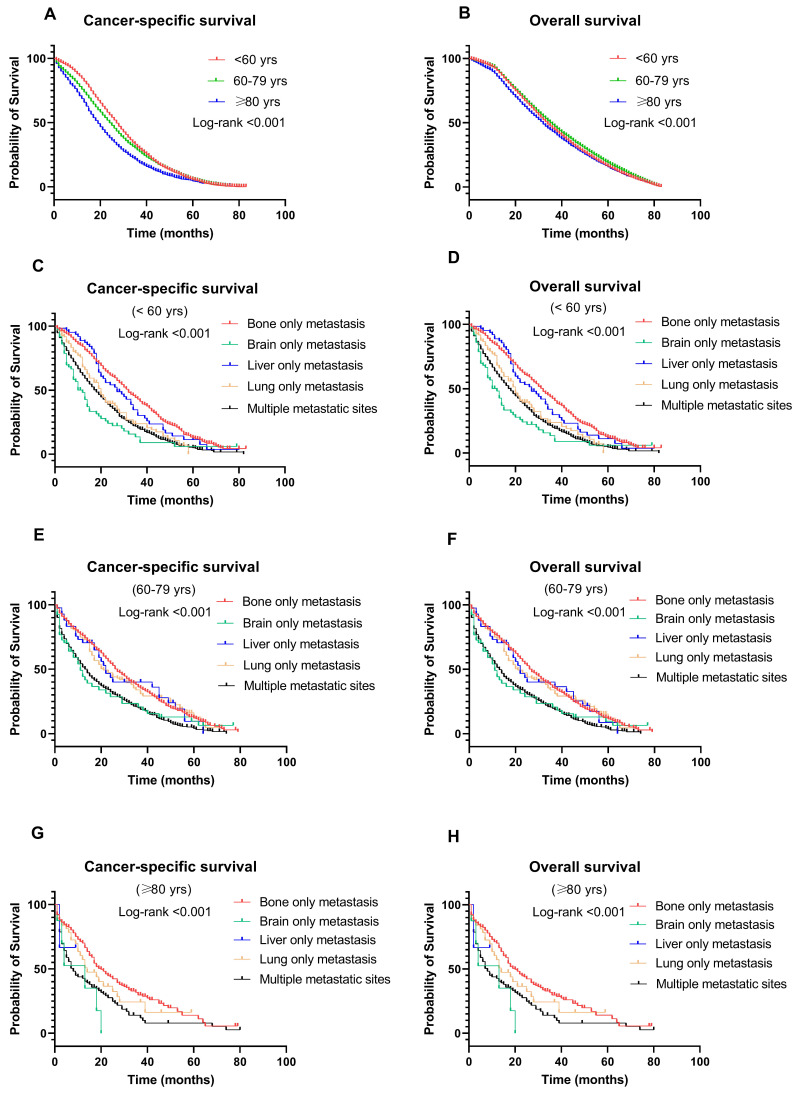
Kaplan-Meier curve of (A) cancer-specific survival and (B) overall survival by age groups among breast cancer patients with metastases; Kaplan-Meier curve of cancer-specific survival and overall survival according to metastasis sites in (C-D) younger (<60 years); (E-F) middle-aged (60-79 years); and (G-H) older (≥ 80 years) patients with breast cancer.

BC-specific survival and OS were compared among the different age groups (Fig. 4A & 4B). The median survival was 32, 36, and 34 months in the older, middle-aged, and younger groups, respectively. Patients in the older group significantly had the worst CSS (OR: 1.402, 95% CI: 1.300-1.512, P < 0.001) and OS (OR: 1.136, 95% CI: 1.095-1.180, P < 0.001). Other factors associated with survival in the multivariate analysis included T stage, N stage, treatment, and metastatic sites (P < 0.001) (Table 2).

### Survival outcomes of different metastasis sites among age groups

Figure 4 shows the CSS and OS of different metastasis sites among age groups. Notably, patients with bone-only metastasis had the best CSS and OS among all age groups (Fig. 4C-H). Meanwhile, in the middle-aged and younger groups, patients with brain-only metastasis had worse CSS and OS compared to other sites of metastasis (median survival time: 9 months and 8 months in CSS, 7 months and 8 months in OS, respectively, P < 0.001). Interestingly, in the older group, liver-only metastasis exhibited a significantly worse CSS and OS than other metastatic patterns.

## Discussion

As the US population ages, the number of older adults with BC is also rising. Nevertheless, only a few studies have focused on older patients, especially those aged ≥ 80 years. Lewis et al. found that only 3% of patients participating in BC clinical trials were aged > 70 years, and the possible reasons for this were low compliance to treatment and a high rate of comorbidities influencing mortality, treatment costs, and expected benefits 11. In the present study, older patients with BC had more distinct clinical presentation and prognosis than younger individuals, which is consistent with previous findings 12,13. In our cohort, we found that several clinicopathological parameters varied significantly among different age groups. Moreover, we observed distinct metastasis patterns in patients aged ≥ 80 years. Our results also show that after adjusting for related parameters, age at diagnosis had a worse independent effect and was a prognostic factor with respect to BC with distant metastases.

There seems to be a debate on distant metastases of BC in different age groups. Yancik et al. showed that older women were more likely to have distal metastases than younger women in a study of 118,000 women with BC 14. However, other investigators have demonstrated that compared to women aged < 65 years, those aged ≥ 65 years were more likely to have early-stage cancers 15. Another study that recruited 243,896 patients showed that in patients with brain or liver metastasis, 64.4% were aged < 65 years. Those with three and four metastasis sites accounted for 65.6% and 73.3%, respectively, of those aged < 65 years, suggesting that they were more likely to have metastasis 16. Regarding the impact of age in this study, we found that older (≥ 80 years) patients had a lower risk of bone, brain, liver, and multiple metastases but a higher risk of lung metastasis, which is consistent with previous findings 17,18. Indeed, the different patterns of distant metastasis in older patients might be due to many factors. One important factor is the time of diagnosis of BC in patients aged ≥ 80 years and the time at which they underwent mammography screening prior to the diagnosis 19. Another factor is the different subtypes of breast carcinoma 20,21. Several previous reports showed that both the HER2 and triple-negative subtypes were significantly associated with brain metastasis, while the HER2 subtype showed a similar incidence of liver metastasis to the luminal B subtype 21,22. Furthermore, Soni et al. found that liver metastases were more frequent in patients with the HER2+ subtype than in those with the luminal B, luminal A, and triple-negative subtypes 23. Comparatively, our results indicate that patients with HER2-enriched BC aged ≥ 80 years had fewer occurrences of brain-only or liver-only metastatic disease 24. The possible reasons for these disparities may be as follows: first, our results should be interpreted cautiously since there were relatively few patients with triple-negative and HER2+ BC in our patient cohort, and second, there were more patients with luminal A subtype of BC in those aged 80 years and older, which is in line with a previous study showing that the group diagnosed at an age above 60 years had a higher percentage of ER/PR-positive BC than patients aged ≤ 60 years 25. Thus, the mechanism explaining the risk of distant metastases in BC patients aged ≥ 80 years remains to be elucidated.

In our analysis, Cox multivariate analyses showed that patients with older age had worse CSS and OS than younger individuals. Indeed, the worst prognosis in the older group (≥ 80 years) was related to poorer physical health and receipt of less aggressive treatment because of age-related deterioration of organ function or comorbidities. A previous study revealed that BC patients aged over 80 years were less likely to receive optimal chemotherapy as their initial treatment than those aged 65 to 75 (11.3% vs. 35.4%) 26. Furthermore, in our population, we found that older patients also had a lower rate of locoregional therapy, such as radiation therapy or surgery, over the entire study period, which is in accordance with previous studies 19,27. Recently, there exists a controversy regarding whether women aged ≥ 80 years benefit from locoregional control as much as younger women, especially in patients that present with distant metastases. Additionally, another explanation might be that potential targeted therapy was associated with improved survival among patients aged < 65 years 28. In fact, several retrospective studies have shown that the benefits and safety of optimal treatment were maintained in patients aged > 75 years 8. Thus, elderly patients still require optimizated care with a view to curative treatment.

Another interesting finding of our study is the different survival outcomes following different patterns of metastasis. To be specific, patients with only bone metastasis had the best CSS and OS in all metastatic patterns among all age groups. The brain-only metastasis group had the poorest outcomes in the middle-aged and younger groups. This might be explained by a low proportion of effective therapy given to patients with brain-only metastasis. Interestingly, for the older group, liver-only metastasis showed significantly worse CSS and OS than the other metastatic patterns. In other words, viable therapeutic alternatives are required for women aged < 60 years with only brain metastases and women aged ≥ 80 years with only liver metastases.

There are some limitations to our study. This was a retrospective study with limited data, and the SEER database provides limited information on systemic treatments, such as endocrine therapy and targeted therapy, which might have influenced survival outcomes. In addition, we were not able to incorporate clinical parameters of patients, such as performance status, details on comorbidities, and the sample size of older women, in our analyses as these data were limited in the SEER database. Despite these limitations, valuable data were provided in this database for analyzing patterns in BC cases with distant metastases across the United States.

## Conclusion

In conclusion, our investigation evidenced novel findings regarding the outcomes of older patients with metastatic BC that women aged ≥ 80 years had a distinctive metastasis pattern, received the least amount of anti-cancer therapy, and had the worst survival outcomes.

Thus, using population-based data from the SEER, our findings summarize the metastasis patterns and survival outcomes of BC patients in three different age groups. The patients aged ≥ 80 years were less likely to be receptive to cancer-related therapy and had the highest rate of cancer mortality among all patients. Therefore, our findings will hopefully help clinicians offer more reasonable modalities of cancer treatment to elderly patients with BC.

## Figures and Tables

**Table 1:** Demographic and clinical characteristics of the cohort

Characteristics	<60 yrs, n=12156 (%)	60-79 yrs, n=19688 (%)	≥ 80 yrs, n=4359 (%)	P-value
**Race**				<0.001
Black	2127 (17.5)	2032 (10.3)	312 (7.4)	
White	8974 (73.8)	16490 (83.8)	3826 (87.8)	
Other ^a^	1055 (8.7)	1166 (5.9)	221 (4.9)	
**Gender**				<0.001
Male	56 (0.5)	159 (0.8)	54 (1.2)	
Female	12100 (99.5)	19529 (99.2)	4305 (98.8)	
**Laterality**				0.188
Left	6184 (50.9)	9844 (50.0)	2245 (51.5)	
Right	5904 (48.6)	9720 (49.4)	2082 (47.8)	
Other ^b^	68 (0.6)	124 (0.6)	32 (0.7)	
**Histology**				<0.001
IDC	9236 (76.0)	13980 (71.0)	2888 (66.3)	
ILC	920 (7.6)	2247 (11.4)	577 (13.2)	
Mix/Other ^c^	2000 (16.5)	3461 (17.6)	894 (20.5)	
**Grade**				<0.001
I	1732 (14.2)	4448 (22.6)	877 (20.1)	
II	3990 (32.8)	8486 (43.1)	1886 (43.3)	
III	5491 (45.2)	5497 (27.9)	1253 (28.7)	
IV	79 (0.6)	56 (0.3)	14 (0.3)	
Unknown	864 (7.1)	1201 (6.1)	329 (7.5)	
**Stage**				<0.001
I	3883 (31.9)	10249 (52.1)	1987 (45.6)	
II	3368 (27.7)	4795 (24.4)	1186 (27.2)	
III	2924 (24.1)	2716 (13.8)	742 (17.0)	
IV	1981 (16.3)	1928 (9.8)	444 (10.2)	
**Intrinsic type**				<0.001
Luminal A	7233 (59.5)	14270 (72.5)	3057 (70.1)	
Luminal B	1234 (10.2)	1439 (7.3)	306 (7.0)	
HER2-enriched	646 (5.3)	632 (3.2)	140 (3.2)	
Triple-negative	2392 (19.7)	2204 (11.2)	526 (12.1)	
Unknown	651 (5.4)	1143 (5.8)	330 (7.6)	
**Surgery**				<0.001
Yes	10616 (87.3)	18037 (91.6)	3775 (86.6)	
No/Unknown	1540 (12.7)	1651 (8.4)	584 (13.4)	
**Radiotherapy**				<0.001
Yes	10828 (89.1)	17576 (89.3)	3491 (80.1)	
No/Unknown	1328 (10.9)	2112 (10.7)	868 (19.9)	
**Chemotherapy**				<0.001
Yes	8193 (67.4)	7179 (36.5)	470 (10.8)	
No/Unknown	3963 (32.6)	12509 (63.5)	3889 (89.2)	

**Notes:**^a^ Other races included American Indians, Asians, and Pacific Islanders; ^b^ Other laterality included paired site, but no information concerning laterality was given; ^c^ included infiltrating duct and lobular carcinoma and infiltrating duct mixed with other types of carcinoma; Abbreviations: IDC, Infiltration Ductal Cancer; ILC, Infiltration Lobular Cancer; Grade I, well differentiated; Grade II, moderately differentiated; Grade III, poorly differentiated; Grade IV, undifferentiated anaplastic.

**Table 2:** Univariate and multivariate analysis of the cancer-specific survival and overall survival of the study population

Characteristics	CSS				OS			
	Univariate		Multivariate		Univariate		Multivariate	
	OR (95% CI)	P-value	OR (95% CI)	P-value	OR (95% CI)	P-value	OR (95% CI)	P-value
**Age**								
<60 yrs	1				1			
60-79 yrs	1.081 (1.036-1.128)	0.000	1.106 (1.058-1.156)	0.000	0.932 (0.911-0.953)	0.000	1.054 (1.029-1.080)	0.000
≥ 80 yrs	1.369 (1.128-1.466)	0.000	1.402 (1.300-1.512)	0.000	1.053 (1.017-1.090)	0.003	1.136 (1.095-1.180)	0.000
**Race**								
Black	1.122 (1.027-1.225)	0.011	1.154 (1.056-1.261)	0.002	1.178 (1.121-1.237)	0.000	1.087 (1.034-1.142)	0.001
White	1.017 (0.940-1.102)	0.671	1.050 (0.970-1.138)	0.288	0.952 (0.913-0.992)	0.019	0.985 (0.945-1.027)	0.485
Other	1		1		1		1	
**Gender**								
Male	1		1		1		1	
Female	1.115 (0.902-1.377)	0.314	1.110 (0.897-1.373)	0.337	0.834 (0.740-0.941)	0.003	0.935 (0.829-1.055)	0.274
**Laterality**								
Left	0.435 (0.366-0.515)	0.000	1.014 (0.829-1.240)	0.890	0.365 (0.320-0.417)	0.000	0.886 (0.764-1.028)	0.111
Right	0.433 (0.365-0.514)	0.000	1.018 (0.833-1.245)	0.861	0.368 (0.323-0.420)	0.000	0.900 (0.776-1.044)	0.166
Other	1		1		1		1	
**Histology**								
IDC	0.832 (0.789-0.876)	0.000	0.941 (0.889-0.995)	0.033	0.958 (0.932-0.984)	0.002	0.986 (0.959-1.014)	0.330
ILC	0.702 (0.644-0.764)	0.000	0.955 (0.873-1.045)	0.315	0.942 (0.905-0.981)	0.004	1.017 (0.976-1.060)	0.421
Mix/Other	1		1		1		1	
**Grade**								
I	1		1		1		1	
II	1.102 (0.999-1.216)	0.052	1.081 (0.977-1.194)	0.132	1.092 (1.062-1.124)	0.000	1.004 (0.975-1.034)	0.769
III	1.448 (1.317-1.591)	0.000	1.440 (1.303-1.591)	0.000	1.405 (1.364-1.447)	0.000	1.158 (1.119-1.199)	0.000
IV	2.016 (1.596-2.547)	0.000	1.805 (1.424-2.288)	0.000	1.464 (1.245-1.722)	0.000	1.138 (0.966-1.340)	0.121
Unknown	2.086 (1.871-2.325)	0.000	1.328 (1.184-1.489)	0.000	1.618 (1.544-1.695)	0.000	1.095 (1.040-1.153)	0.001
**T stage**								
T0	1		1		1		1	
T1	0.394 (0.313-0.495)	0.000	0.751 (0.579-0.974)	0.031	0.574 (0.502-0.655)	0.000	1.191 (1.028-1.380)	0.020
T2	0.467 (0.373-0.586)	0.000	0.832 (0.644-1.076)	0.162	0.737 (0.644-0.842)	0.000	1.357 (1.172-1.571)	0.000
T3	0.519 (0.413-0.652)	0.000	0.922 (0.712-1.194)	0.539	0.859 (0.749-0.985)	0.029	1.494 (1.287-1.734)	0.000
T4	0.704 (0.561-0.884)	0.002	0.991 (0.767-1.280)	0.943	1.280 (1.116-1.468)	0.000	1.728 (1.491-2.003)	0.000
TX	0.940 (0.738-1.196)	0.612	0.900 (0.695-1.165)	0.424	1.638 (1.408-1.907)	0.000	1.382 (1.181-1.617)	0.000
**N stage**								
N0	1		1		1		1	
N1	1.134 (1.078-1.194)	0.000	1.056 (1.000-1.116)	0.049	1.278 (1.246-1.310)	0.000	1.097 (1.066-1.128)	0.000
N2	1.037 (0.974-1.103)	0.254	1.082 (1.013-1.156)	0.020	1.312 (1.263-1.362)	0.000	1.126 (1.080-1.174)	0.000
N3	1.164 (1.094-1.240)	0.000	1.133 (1.059-1.213)	0.000	1.540 (1.478-1.604)	0.000	1.205 (1.151-1.261)	0.000
NX	2.416 (2.151-2.713)	0.000	1.356 (1.190-1.545)	0.000	3.227 (2.941-3.540)	0.000	1.322 (1.191-1.467)	0.000
**Intrinsic type**								
Luminal A	0.632 (0.584-0.684)	0.000	0.808 (0.745-0.877)	0.000	0.902 (0.863-0.943)	0.000	1.035 (0.989-1.083)	0.143
Luminal B	0.693 (0.629-0.765)	0.000	0.769 (0.695-0.851)	0.000	1.012 (0.957-1.070)	0.667	1.032 (0.974-1.092)	0.285
HER2-enriched	0.781 (0.702-0.868)	0.000	0.933 (0.836-1.041)	0.216	1.158 (1.083-1.238)	0.000	1.146 (1.070-1.228)	0.000
Triple-negative	0.967 (0.890-1.051)	0.435	1.372 (1.256-1.500)	0.000	1.304 (1.240-1.372)	0.000	1.425 (1.351-1.503)	0.000
Unknown	1		1		1		1	
**Surgery**								
Yes	0.445 (0.425-0.466)	0.000	0.581 (0.544-0.620)	0.000	0.350 (0.338-0.362)	0.000	0.569 (0.542-0.598)	0.000
No/Unknown	1		1		1		1	
**Radiotherapy**								
Yes	0.788 (0.742-0.836)	0.000	0.833 (0.783-0.887)	0.000	0.772 (0.748-0.797)	0.000	0.821 (0.794-0.848)	0.000
No/Unknown	1		1		1		1	
**Chemotherapy**								
Yes	0.842 (0.807-0.879)	0.000	0.883 (0.837-0.930)	0.000	1.131 (1.108-1.155)	0.000	0.895 (0.871-0.920)	0.000
No/Unknown	1		1		1		1	
**Sites of metastasis**								
No	1		1		1		1	
Bone only	1.436 (1.348-1.529)	0.000	1.168 (1.084-1.259)	0.000	2.057 (1.956-2.163)	0.000	1.333 (1.257-1.414)	0.000
Brain only	3.285 (2.711-3.982)	0.000	2.200 (1.799-2.688)	0.000	4.314 (3.652-5.097)	0.000	2.531 (2.130-3.007)	0.000
Liver only	1.441 (1.162-1.787)	0.001	1.337 (1.076-1.662)	0.009	2.123 (1.763-2.555)	0.000	1.500 (1.245-1.809)	0.000
Lung only	2.053 (1.775-2.375)	0.000	1.352 (1.163-1.572)	0.000	2.775 (2.451-3.142)	0.000	1.573 (1.384-1.787)	0.000
Multiple	2.443 (2.302-2.593)	0.000	1.816 (1.686-1.957)	0.000	3.651 (3.473-3.839)	0.000	2.072 (1.946-2.206)	0.000

OS, overall survival; CSS, cancer-specific survival; IDC, Infiltration Ductal Cancer; ILC, Infiltration Lobular Cancer; Grade I, well differentiated; Grade II, moderately differentiated; Grade III, poorly differentiated; Grade IV, undifferentiated anaplastic; OR, odds ratio; CI, confidence interval.

## Abbreviations

BC: breast cancer
OS: overall survival
CSS: cancer-specific survival
SEER: Surveillance, Epidemiology, and End Results
